# Why doesn’t high cholesterol hurt?

**DOI:** 10.18632/aging.102620

**Published:** 2019-12-11

**Authors:** Julius L. Katzmann, Ulrich Laufs

**Affiliations:** 1 Klinik und Poliklinik für Kardiologie, Universitätsklinikum Leipzig, Leipzig, Germany

**Keywords:** hypercholesterolemia, evolution, LDL cholesterol, cardiovascular disease, atherosclerosis

Touching a hotplate after usage is an experience which one intuitively will try to avoid in future. Falling from a bike and breaking an arm will prompt the affected to avoid unnecessary movements of the limb to relieve the pain. After starving a whole day, one will usually get hungry and attempt to have a meal. Being physically exhausted, one is usually keen to get some rest.

These are examples of physiological functions which developed during evolution and are still meaningful in today’s life: To avoid any kind of potential harm to the body, to restore physiological functions when out of balance, and to satisfy the biochemical needs of the organism by giving itself signals that favour respective behaviour. Acknowledging this, one may ask why causal drivers of cardiovascular disease, which is the most common cause of death in Western societies, do not prompt the individual to behave in a way that diminishes these risk factors. Why does the insult to the vascular endothelium by smoking, high blood pressure, or high blood sugar not cause discomfort? Why does the vasculature of a person with familial hypercholesterolemia not hurt? Why does a person with high cholesterol not feel antipathy for fatty and high-caloric meals?

From an evolutionary perspective, the mentioned physiological functions preserve the integrity of the body with the ultimate goal to enable the organism to reproduce. Physical harms and unsatisfied physiological needs directly affect the probability of reproductive success and therefore, individuals displaying favourable behaviour in this regard are more likely to pass their genes on to the next generation. Any genetic trait with effects that become relevant only after reproduction does not exert pressure to be sustained. Such traits may even have been beneficial under the circumstances of feast-famine cycles under which they evolved. This explanation why detrimental genetic traits leading to hypercholesterolemia, diabetes mellitus, and obesity, occur with such high prevalence has been called “thrifty gene hypothesis” [1].

**Figure 1:**
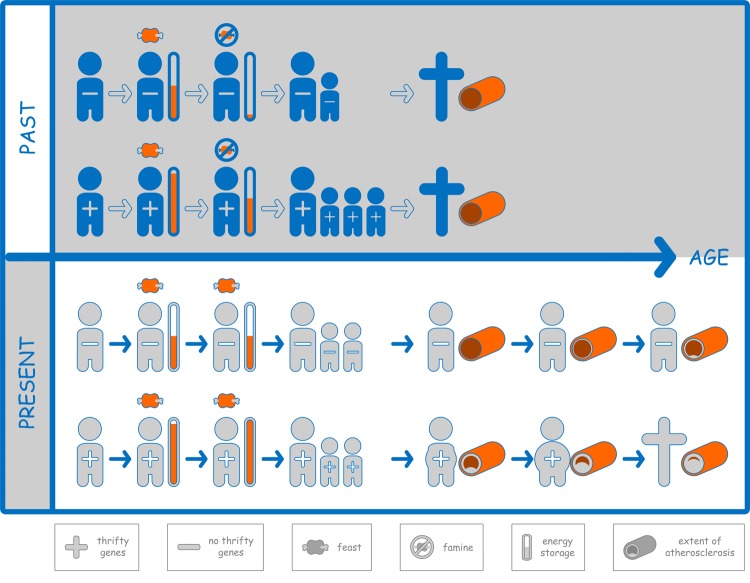
“+” denotes the presence of so-called thrifty genes like those leading to hypercholesterolemia, “–“ denotes the absence of these genes. In the past (upper panel), carriers of thrifty genes were able to build comparably larger amounts of energy storage (orange bars) after feast episodes. In famine episodes, carriers of thrifty genes were therefore more likely to reproduce. Usually, people died before the negative consequences of metabolic profiles associated with thrifty genes became relevant. In contrast, today (lower panel), in the absence of famines, thrifty genes do not become relevant with respect to reproduction, but lead to traits of the metabolic syndrome, atherosclerosis, and earlier death due to atherosclerotic cardiovascular disease as the most common cause of death in Western societies.

We discussed this hypothesis focusing on the high prevalence of hypercholesterolemia in a recent article [2]. Hypercholesterolemia affects one in two individuals in Western societies and is, relying on different lines of evidence, causal for the development of atherosclerotic cardiovascular disease [3]. Genetic traits that favour high blood levels of cholesterol have likely been beneficial long ago to foster energy security and in consequence, lead to early reproduction (Figure 1). It is indisputable that cholesterol is an essential element of the human body, but with 93% of all cholesterol being intracellular and famine episodes being virtually absent today, do we than still need *any* cholesterol in our bloodstream? Very low levels of LDL cholesterol, due to mutations or aggressive medical treatment, do not appear to have any detrimental effects. And even the severely impaired ability for LDL cholesterol uptake in peripheral cells from blood as can be found in homozygous familial hypercholesterolemia with defective LDL receptors is not associated with developmental problems, vitamin deficiency, or central nervous dysfunction [2].

We propose that high blood cholesterol is a remnant of evolution that has detrimental effects with increasing age. High plasma cholesterol should therefore be a target of early lifestyle and medical interventions. Early measures to reduce LDL cholesterol at a population-wide level therefore have the potential to dramatically reduce the occurrence of cardiovascular disease [4].

An additional contributor to high plasma cholesterol levels is the cholesterol contained by lipoprotein(a). Lipoprotein(a) levels are inherited to an even higher degree than those of LDL cholesterol. It is poorly understood why high lipoprotein(a) levels were preserved during evolution, and what the physiological functions of lipoprotein(a) are. However, the causal role of lipoprotein(a) in the development of atherosclerosis is clear. With elevated lipoprotein(a) levels that were calculated to confer a comparable cardiovascular risk as having heterozygous familial hypercholesterolemia but with twice the prevalence [5], outcome trials on the effects of selective and potent lipoprotein(a) lowering are eagerly awaited.

The first steps towards diminishing the genetic burden of hypercholesterolemia have been laid out in the recently published European guidelines on dyslipidemia [6]: There is virtually no lower limit of LDL cholesterol levels; lower LDL cholesterol goals than ever have been recommended; and the general screening on lipoprotein(a) elevation has been introduced.

Finally, while the question why hypercholesterolemia does not hurt may primarily be of academic interest, the answer provided may be useful for patient care as well. It can explain why cholesterol levels referred to as “normal” by patients and physicians is still associated with subclinical atherosclerosis as precursor of established cardiovascular disease [7] and should be a target of treatment. Since high cholesterol does not hurt, lipid lowering will not confer symptomatic benefit. Therefore, patient discussion – including the principles discussed here – is the key to medication adherence. As with accumulating evidence of the cumulative effects of hypercholesterolemia, treatment tends to be more intensive and to start earlier in life, potentially leading to a lifelong pharmacological treatment beginning in adolescence. The rationale of a therapy directed at a disease that manifests decades ahead may be easier to understand when comprehending it as an opportunity to counteract genetic bad luck.
